# Tricuspid Valve Endocarditis: A Disguise In Multifocal Septic Arthritis

**DOI:** 10.7759/cureus.11375

**Published:** 2020-11-08

**Authors:** Zain U Abideen, Rehman M Bhatti, Farhan Khalid, Ali Jaan, Zahoor Ahmed

**Affiliations:** 1 Internal Medicine, Abington Memorial Hospital, Jefferson Health, Abington, USA; 2 Internal Medicine, Ayub Medical College, Abbottabad, PAK; 3 Internal Medicine, King Edward Medical University, Mayo Hospital, Lahore, PAK

**Keywords:** tricuspid valve endocarditis, septic arthritis, case report

## Abstract

Infective endocarditis (IE) is a rare cause of multifocal septic arthritis. We report a patient who presented with oligo-articular septic arthritis as a complication of tricuspid valve endocarditis, a rare condition. A 69-year-old female presented to the emergency department with complaints of fever, myalgia, right shoulder, and right knee pain. Initial investigation elaborated on elevated C-reactive protein, erythrocyte sedimentation rate (ESR), and white blood cell. Imaging studies, including X-ray, were unrevealing. Blood cultures came out positive for Methicillin-sensitive Staphylococcus aureus (MSSA) bacteremia. Magnetic resonance imaging (MRI) of the right shoulder and right knee showed joint effusion with soft tissue swelling. Diagnostic arthroscopic washout of right shoulder and right knee showed neutrophilic pleocytosis, and the cultures were positive for MSSA. Despite adequate antibiotic coverage for MSSA bacteremia with oligo-articular/multifocal septic arthritis, the patient's fever did not resolve. Initial transthoracic echocardiography (TTE) was negative for any vegetation. Transesophageal echocardiography (TEE) showed vegetations on the tricuspid valve with tricuspid regurgitation and was diagnosed with acute bacterial IE. A multi-disciplinary approach was adopted. She underwent tricuspid valve repair with annuloplasty and was treated with nafcillin for six weeks. She successfully recovered from MSSA bacteremia, and follow-up TEE was negative for any vegetations.

## Introduction

Infective endocarditis (IE) is commonly seen in intravenous drug abusers. The predisposing risk factors like immune suppression, central venous access device, prosthetic heart valves, and unrepaired cyanotic heart disease also increase the risk of the disease [1]. The stigmata of IE include post-infectious glomerulonephritis, peripheral septic emboli presenting as stroke, pulmonary emboli with infarction, and splenic infarction. It also involves Janeway lesions, Roth spots, and Osler nodes. These stigmata of IE raises the suspicion of IE as the underlying etiology. However, multifocal oligo-articular septic arthritis involving shoulder and knee joint is a rare manifestation and complication of IE.

## Case presentation

**Figure 1:**
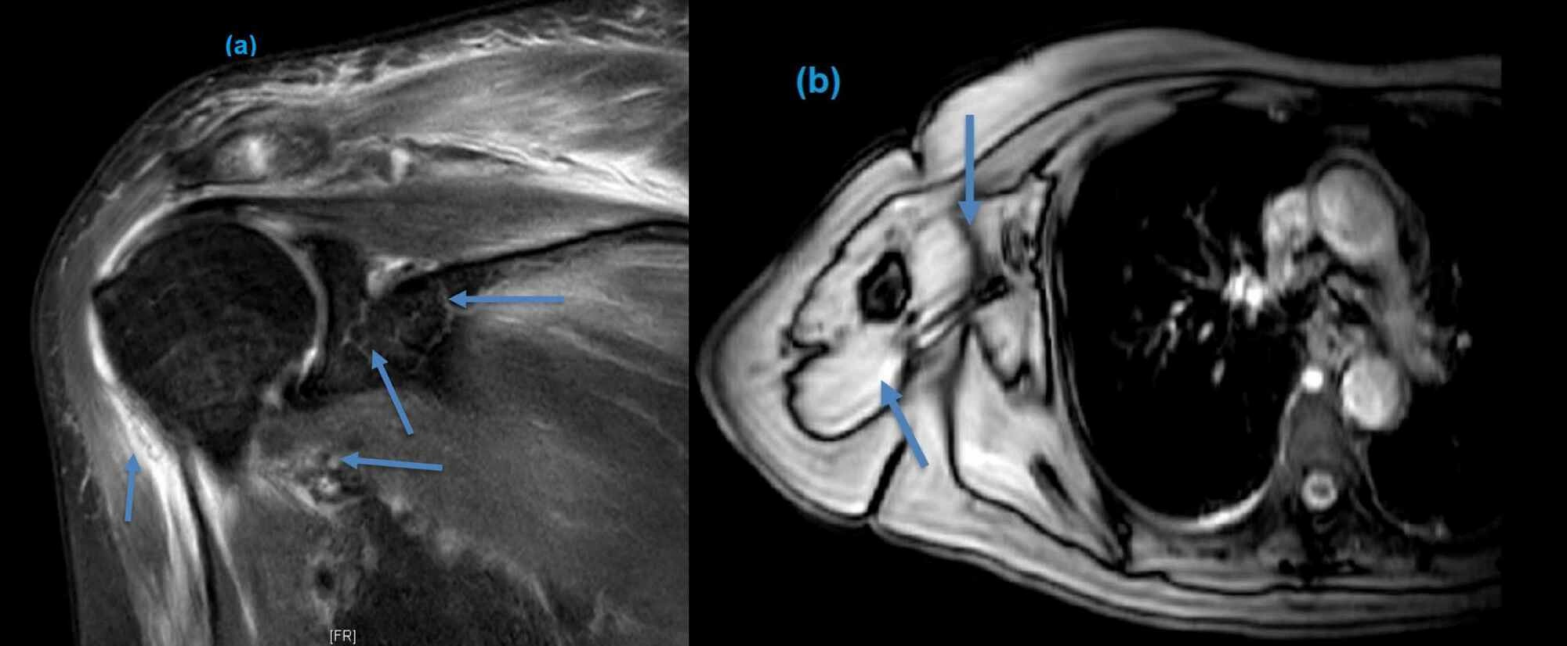
Magnetic resonance imaging (MRI) of the right shoulder showing joint effusion extending up to sub-coracoid space and proximal humerus associated with soft tissue edema (blue arrow) in coronal (a) and axial (b) view.

A 69-year-old female with a past medical history of hypothyroidism and recently diagnosed anal carcinoma (on chemotherapy and radiotherapy) presented with complaints of high-grade fever, myalgia, and joint pains limited initially to the right knee joint and right shoulder joint for the last five days. The patient recently had placement of the port-a-cath for chemotherapy one month ago. Clinical examination revealed right shoulder joint swelling, redness, and tenderness to palpation along with right knee joint swelling. The limb was neurovascular intact. The port-a-cath site was also erythematous consistent with localized cellulitis. Initial investigation revealed leukocytosis, normocytic normochromic anemia, elevated C-reactive protein, erythrocyte sedimentation rate (ESR), and mild lactic acidosis consistent with sepsis. Radiographs of the right shoulder joint and right knee joint showed soft tissue swelling with no fracture. Blood cultures came positive with Methicillin-sensitive Staphylococcus aureus (MSSA). Initially, she was started on vancomycin for sepsis secondary to MSSA before sensitivities. After confirmation of MSSA, she was commenced on cefazolin. Initial transthoracic echocardiography (TTE) was nonsignificant without any vegetation. The magnetic resonance imaging (MRI) of the right upper extremity showed right shoulder joint effusion extending up to sub-coracoid space and proximal humerus associated with soft tissue edema (Figure 1).

Subsequently, the patient underwent arthroscopic washout of right glenohumeral joint effusion and right knee under anesthesia, and cultures/sensitivity came positive with MSSA consistent with a diagnosis of right shoulder and right knee joint septic arthritis.

Repeat cultures after three days showed persistent MSSA despite on cefazolin. During hospitalization, the patient developed acute hypoxic respiratory failure, and the patient was shifted to the medical intensive care unit. Computed tomography of the chest showed multiple pulmonary nodules raising the suspicion of septic emboli in the context of sepsis secondary to MSSA and septic arthritis. Due to persistent bacteremia, transesophageal echocardiography (TEE) was planned, which revealed moderate-sized vegetation on the tricuspid valve associated with tricuspid regurgitation. The patient was diagnosed with tricuspid endocarditis in the context of positive MSSA. A multidisciplinary approach involving a cardiologist, cardiovascular surgeon, and infectious disease specialist was adopted. The patient underwent tricuspid valve repair with annuloplasty and replacement of a septal leaflet with the pericardium. The patient was continued on nafcillin for six weeks, which resulted in the resolution of symptoms.

## Discussion

Septic arthritis usually presents as a hot, tender, and swollen joint or multiple joints. The hip and knee joints are the most commonly affected joints [2]. However, shoulder joint involvement is rare. Musculoskeletal symptoms and complications of IE have been reported in many studies. The arthralgias and lower back pain were common in these patients, though the documented infection was uncommon. These infections are most likely associated with intravenous drug abusers [3].

In IE, bacteria cause septic arthritis presenting with a joint's inflammation, typically resulting in the signs and symptoms of acute monoarticular septic arthritis or oligo-articular septic arthritis. A definite diagnosis of IE is based on Duke clinical criteria: two major or one major including three minor or five minor criteria. Major criteria involve two positive blood cultures with the typical organism (Streptococcus viridans, Streptococcus bovis, Streptococcus aureus, or Enterococcus) or three positive blood cultures out of three or four, and echocardiography without vegetation of definitive mass or new valve regurgitation. Minor criteria incudes any predisposing cardiac condition, fever ≥ 38^O^C, vascular phenomenon, immune phenomenon, blood cultures not meeting the above criteria, or echocardiographic valve abnormality but not diagnostic for vegetation [4].

Bacterial IE is considered as a differential diagnosis if some of the clinical manifestations of IE are present but are not enough to meet definite IE criteria. Most of the patients are treated empirically because of the critical consequences of the disease. IE can be acute or subacute based on the clinical presentation of the patient. The patient may present with high fever, significant systemic toxicity (acute bacterial IE), or others may present with non-specific symptoms like fatigue, malaise, joint pain, anorexia, and mild fever lasting days to weeks (subacute IE). The patients with IE may also present with severe localized infections such as cellulitis or septic arthritis, as in our case.

The patients having predisposing conditions or with high-risk behavior are more prone to have bacterial IE. Physical findings may involve splenomegaly, petechiae, signs of heart dysfunction, or retinal/conjunctival hemorrhages. Serum and blood chemistry may include anemia, microscopic hematuria, and elevated ESR. However, bacterial IE can occur in the absence of many of these manifestations [5]. IE's definitive diagnosis is based on history, physical exam findings, and initial laboratory findings, with a minimum of two sets of blood cultures, if possible, 12 hours apart. An echocardiogram is preferred because most of the cardiac lesions are manifested on TTE. If the results of TTE are negative, TEE may be performed due to high specificity [4].

Our case report highlights a rare manifestation of oligo-articular septic arthritis, particularly involving shoulder joint as a complication of IE. This case particularly emphasizes the significance of clinical examination and a high index of IE suspicion with septic arthritis involving axial joints. This case also highlights the importance of early blood cultures and TEE's implementation in a patient with persistent bacteremia.

## Conclusions

A multidisciplinary approach is a necessity for the accurate diagnosis and appropriate management of complicated and unique cases. Early involvement of physicians from different specialties and composing a team for the early diagnosis and effective treatment on time can prevent morbidity and mortality. IE is a potentially lethal condition, and it can lead to fatal consequences if not diagnosed and managed in a timely and proper manner. Due to its variable clinical presentations, a high index of clinical suspicion is required to diagnose IE. IE is a medical emergency and should be considered a differential diagnosis for any patient with septic arthritis and persistent bacteremia.
